# Germline *TP53* alterations in Finnish breast cancer families are rare and occur at conserved mutation-prone sites

**DOI:** 10.1054/bjoc.2000.1530

**Published:** 2001-01-01

**Authors:** K Rapakko, M Allinen, K Syrjäkoski, P Vahteristo, P Huusko, K Vähäkangas, H Eerola, T Kainu, O-P Kallioniemi, H Nevanlinna, R Winqvist

**Affiliations:** Departments of Clinical Genetics, Oncology and Radiotherapy, and Pharmacology and Toxicology, University of Oulu/Oulu University Hospital, Oulu, Finland; Laboratory of Cancer Genetics, Institute of Medical Technology, Tampere University Hospital, Tampere, Finland; Departments of Obstetrics and Gynaecology, Oncology, Helsinki University Central Hospital, Helsinki, Finland

**Keywords:** hereditary breast cancer, *TP53* mutations, Li-Fraumeni syndrome

## Abstract

We have screened for germline *TP53* mutations in Finnish *BRCA1* and *BRCA2* mutation-negative families. This study represents the largest survey of the entire protein-encoding portion of *TP53*, and indicates that mutations are only found at conserved domains in breast cancer families also meeting the criteria for Li-Fraumeni/Li-Fraumeni-like syndrome, explaining only a very small additional fraction of the hereditary breast cancer cases. © 2001 Cancer Research Campaign http://www.bjcancer.com


*British Journal of Cancer* (2001) **84**(1), 116–119
© 2001 Cancer Research Campaign
doi: 10.1054/ bjoc.2000.1530, available online at http://www.idealibrary.com on IDEAL®

http://www.bjcancer.com

Short Communication

### Germline *TP53* alterations in Finnish breast cancer families are rare and occur at conserved mutation-prone sites

**K Rapakko[1*], M Allinen[1*], K Syrjäkoski[2], P Vahteristo[3], P Huusko[1], K Vähäkangas[4], H Eerola[3,5], T Kainu[2], O-P Kallioniemi[2], H Nevanlinna[3] and R Winqvist[1,6]**

Departments of [1]Clinical Genetics, [6]Oncology and Radiotherapy, and [4]Pharmacology and Toxicology, University of Oulu/Oulu University Hospital, Oulu, Finland; [2]Laboratory of Cancer Genetics, Institute of Medical Technology, Tampere University Hospital, Tampere, Finland; Departments of [3]Obstetrics and Gynaecology, and [5]Oncology, Helsinki University Central Hospital, Helsinki, Finland

**Summary** We have screened for germline *TP53* mutations in Finnish *BRCA1* and *BRCA2* mutation-negative families. This study represents the largest survey of the entire protein-encoding portion of *TP53*, and indicates that mutations are only found at conserved domains in breast cancer families also meeting the criteria for Li-Fraumeni/Li-Fraumeni-like syndrome, explaining only a very small additional fraction of the hereditary breast cancer cases. © 2001 Cancer Research Campaign http://www.bjcancer.com

**Keywords:** hereditary breast cancer; *TP53* mutations; Li-Fraumeni syndrome

Approximately 5–10% of breast cancer patients have some degree of family history and may be carriers of an inherited susceptibility to the disease (Claus et al, 1996). However, mutations in the two major breast cancer susceptibility genes, *BRCA1* and *BRCA2* (Miki et al, 1994; Wooster et al, 1995), have been found only in about 20% of Finnish high-risk breast cancer families (Vehmanen et al, 1997a, b; Huusko et al, 1998). Very recently, the chromosomal location of a new susceptibility gene was identified (Kainu et al, 2000). This and other genes, such as *TP53*, *PTEN*, and possibly *ATM*, are responsible for an additional fraction of breast cancer predisposition (reviewed in Easton, 1999).

Somatic mutations in the *TP53* gene are found in almost all kinds of tumours (Hollstein et al, 1991). Mutations are usually clustered within the most conserved regions of exons 4, 5, 7 and 8, corresponding to the sequence-specific DNA binding domain of the protein (Levine, 1997). Germline *TP53* mutations have been found in patients with the rare Li-Fraumeni syndrome (LFS), characterized by breast cancer, osteosarcoma, leukaemia, brain and adrenocortical tumours at an early age (Birch et al, 1994; Varley et al, 1997b; reviewed in Eng et al, 1997 and Varley et al, 1997a). A similar cancer phenotype has recently been observed in patients carrying hereditary mutations in the checkpoint kinase gene, *CHK2*, indicating genetic heterogeneity within LFS (Bell et al, 1999).

In the previous study by Huusko et al (1999), we screened 7 Finnish LFS and Li-Fraumeni-like (LFL) families for *TP53* exon 5–8 mutations. Two changes were identified (Tyr220Cys and Asn235Ser), both of which appeared to associate with accumulation of female breast cancer. Based on these results, we wanted to see whether germline *TP53* mutations could also explain familial breast cancer cases without LFS/LFL phenotype and if mutations could be found outside the conserved segment. We also anticipated finding geographical clustering, because of the strong founder effects regarding *BRCA1* and *BRCA2* mutations in Finland (Huusko et al, 1998; Sarantaus et al, 2000). Here, a large cohort of 130 breast cancer patients from 108 Finnish *BRCA1* and *BRCA2* mutation-negative breast cancer families were screened for *TP53* germline alterations covering the entire protein encoding region (exons 2–11) of the gene.

We analysed 130 subjects from 108 Finnish *BRCA1* and *BRCA2* mutation-negative breast cancer families (Vehmanen et al, 1997a, b; Huusko et al, 1998). Of these families, 5 had previously been studied for *TP53* exon 5–8 mutations (Huusko et al, 1999). Geographically, the families originated from 3 regions of Finland: 79 from the Oulu University Hospital area, 13 from the Tampere University Hospital area and 16 from the Helsinki University Central Hospital area. The criteria for inclusion were 3 or more cases of breast cancer in first- or second-degree relatives, early disease onset (≤35 years), bilateral breast cancer, or multiple tumours including breast cancer in the same individual. 75 families met the criteria for moderate- to high-risk hereditary breast cancer only, 32 for both hereditary breast cancer and LFL, and 1 for both hereditary breast cancer and LFS (Table 1; Birch et al, 1994; Eng et al, 1997). To search for founder effects, additional *BRCA1* and *BRCA2* mutation-negative breast cancer families (50 for Tyr220Cys and Asn235Ser, and 5 for Arg248Gln), originating from the same geographical regions as those with the identified mutations, were analysed. In addition, DNAs from 500 unselected consecutive breast cancer patients from the Tampere region were used to establish the mutation frequency in the population. Informed consent to obtain pedigree data and a blood specimen for the study was obtained from all patients. The Ethical Boards of the participating hospitals and the Finnish Ministry of Social Affairs and Health have approved the study.

*Received 5 July 2000*
*Revised 15 September 2000*
*Accepted 19 September 2000*

*Correspondence to:* R Winqvist

*Equal contribution

**Table 1** Summary classification of all studied 108 cancer families according to occurrence of breast, other LFS/LFL-associated (osteosarcoma, leukaemia, brain or adenocortical tumours) and other cancers in 1st and 2nd degree relatives

| **No. of breast cancer cases within a family** | **No. of other cancers within a family** | | | | | | | **No. of LFS/LFL-associated cancers within a family** |
|---|---|---|---|---|---|---|---|---|
| | 0 | 1 | 2 | 3 | 4 | 5 | 6 | |
| 1 | **1** | **2** | **1** | **2** | | **1** | | 0 |
| | | **1** | | | | | | 1 |
| | | **1** | | | | | | 2 |
| | | | | | | | | 3 |
| | | | | | | | | 4 |
| 2 | **6** | **9** | **9** | | **1** | | **1** | 0 |
| | **2** | **3** | **1** | **1** | | | | 1 |
| | | | | **1** | **1** | | | 2 |
| | | | | | | | | 3 |
| | | | | | | | | 4 |
| 3 | **5** | **5** | **10** | **1** | **2** | **1** | **3** | 0 |
| | **1** | **3** | **4** | **2** | **1** | | | 1 |
| | **1** | | | | | | | 2 |
| | | | | | | | | 3 |
| | | | | | | | | 4 |
| 4 | **1** | **1** | **3** | **1** | **1** | | | 0 |
| | | **2** | **1** | | **1** | | **1** | 1 |
| | | | **1** | | | | | 2 |
| | **1** | | | | | | | 3 |
| | | | | | | | | 4 |
| 5 | **1** | **2** | **1** | | **1** | | | 0 |
| | | | | | | | | 1 |
| | | | | | | | **1** | 2 |
| | | | | | | | | 3 |
| | | | | | | | | 4 |
| ≥6 | **1** | | | | **1** | | | 0 |
| | | | | | | | | 1 |
| | | | | **1** | | | | 2 |
| | | | | | | | | 3 |
| | | | | | | | | 4 |
| Total no. of families: 108 | | | | | | | | |

Numbers in bold indicate the total number of families in each class. Of the 130 breast cancer cases, 12 (9%) were identified at or below age 35, 86 (66%) between ages 36–60, and 32 (25%) at or above age 61.

Using genomic DNA, *TP53* exons 2–11 were screened by conformation sensitive gel electrophoresis (CSGE) or fluorescense-CSGE as described earlier (Ganguly et al, 1998; Huusko et al, 1998). Samples with a band shift were sequenced as instructed by the apparatus manufacturer Li-Cor (Lincoln, USA). DNAs from 500 unselected consecutive breast cancer cases were screened using DNA minisequencing (Syvänen, 1999). Oligonucleotide sequences were designed based on GenBank sequence information (U94788).

In the present study, 108 breast cancer families were screened for *TP53* mutations. This is a considerably larger number of families fulfilling the criteria of hereditary breast cancer than in any previous study. The CSGE and F-CSGE analyses of exons 2–11 revealed a constitutional *TP53* alteration, Arg248Gln, in one of the studied families. In addition, one silent variant (Arg213Arg) and other known polymorphisms in the intronic region between the exons 2 and 3, as well as in exon 4, were detected (data not shown).

The subject with the Arg248Gln (CGG→CAG) mutation showed strong family history of breast cancer. The cancer spectrum of this family (#6001, Figure 1A) also matches the LFL criteria (Birch et al, 1994; Eng et al, 1997). Immunohistochemical analysis results from patient records revealed positive p53 staining in the tumours of all three of the mutation carriers.

Arg248 resides in the highly conserved region of exon 7 and is the most frequently altered residue of the p53 protein, being essential for DNA binding functions (Cho et al, 1994). Therefore, the Arg248Gln substitution leads to defective contacts with target DNA. Agr248Gln has most frequently been detected in colorectal and breast tumours (Lasky and Silbergeld, 1996). It is also the most commonly found alteration in LFS (Shibata et al, 1996). In the family carrying Arg248Gln (Figure 1A), we were unable to determine the mutational status of the proband's (case 19) paternal grandparents. Since they both died at an old age without evidence of cancer, it is possible that this is a de novo mutation.

Screening of the Arg248Gln mutation and the previously detected two Finnish germline *TP53* mutations (Tyr220Cys, Asn235Ser; Huusko et al, 1999) was expanded to include 500 unselected consecutive breast cancer cases. No mutations were found, suggesting that the frequency of these mutations is very low in the general breast cancer population. Our results support the observation that *TP53* alterations are rare and explain only a negligible fraction of breast cancer cases at the population level, mainly associated with strong family history of breast cancer. There was no evidence of founder effects that otherwise are common in hereditary diseases in Finland. This may be due to the rareness/young age/more severe effect on survival of the studied *TP53* mutations, as compared to the situation in for instance HNPCC- (Nyström-Lahti et al, 1995) and *BRCA1/BRCA2*-related cancer predisposition (Huusko et al, 1998; Sarantaus et al, 2000). Evidence against founder effects was also obtained by studying additional breast cancer cases originating from the same geographical areas as those with mutations.

Taking together the two studies, we have analysed the entire *TP53* protein-encoding region in 108 breast cancer families and found mutations in three (2.8%) of them (Figure 1). This frequency is in agreement with the concept that mutations preferentially occur in LFS/LFL-related patients. Zeleda-Hedman et al (1997), Warren et al (1992), Prosser et al (1991) and Patel et al (1995) studied 109, 25, 5 and 4 breast cancer families, respectively, without finding any mutations. Boerresen et al (1992) studied 237 women with breast cancer, of which 30 had at least one first-degree relative with breast cancer, 40 had breast cancer before age 35, and 167 represented unselected breast cancer patients. Only one unselected and one early-onset patient were found to carry a mutation. On more detailed review, both of these cases had a family history of breast cancer and other malignancies suggestive of LFS. Lidereau and Soussi (1992) studied 19 unrelated cases with bilateral breast cancer, but no *TP53* mutations were detected. Breast and other cancers had occurred in the relatives of 7 of the 12. Sidransky et al (1992) found 1 of 126 patients with early-onset breast cancer carrying a germline change. These earlier mutation analyses have usually been limited to exons 5–8. Due to recent observations that the mutations could also reside in domains responsible for transcription and oligomerization control (exons 2–4 and 9–11) (Varley et al, 1997a), we decided to investigate these regions too. However, our results support the existing view (Toguchida et al, 1992; Birch et al, 1994; Cornelis et al, 1997; Varley et al, 1997b), suggesting that the breast cancer

© 2001 Cancer Research Campaign 

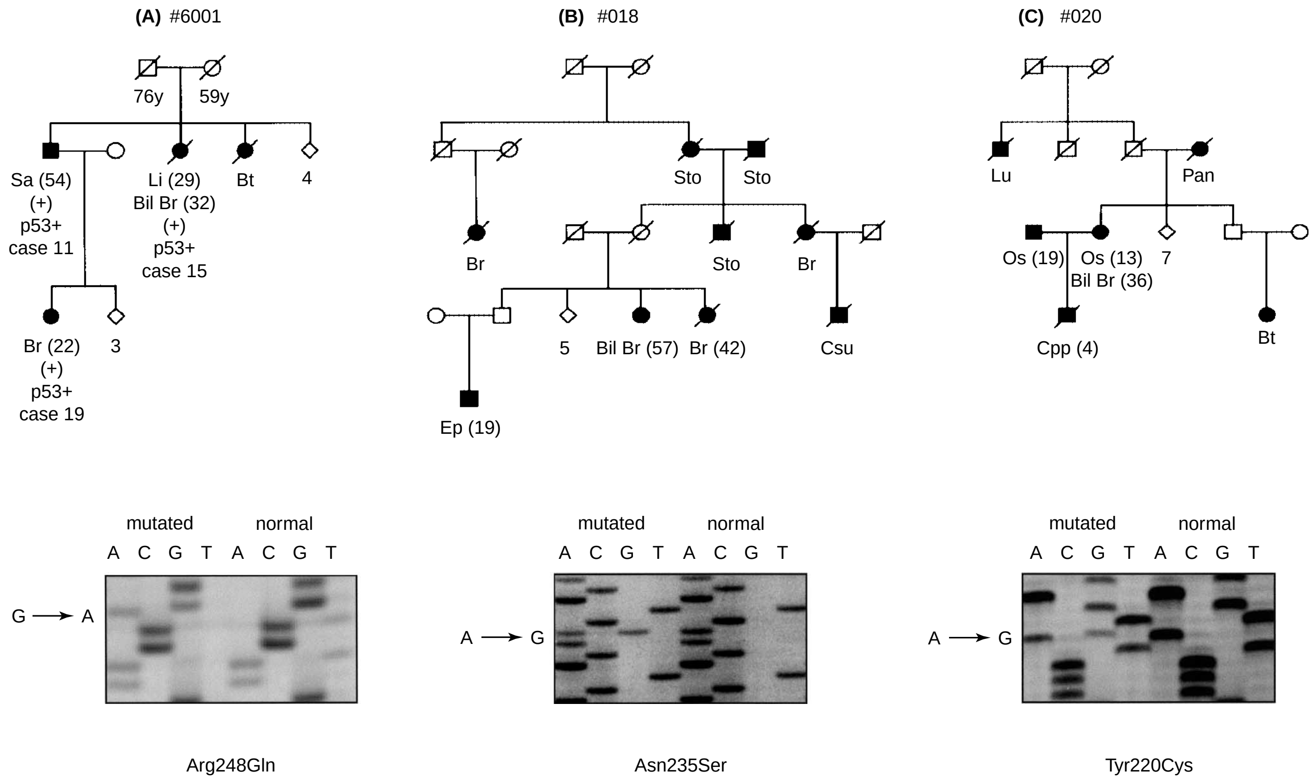

**Figure 1** Summary of the identified three Finnish families with germline *TP53* mutations: (**A**) Family #6001 Arg248Gln, (**B**) Family #018 Asn235Ser, and (**C**) Family #020 Tyr220Cys. Tumours: Bil Br, bilateral breast; Br, breast; Bt, brain; Cpp, choroid plexus papilloma; Csu, cancer site unknown; Ep, ependyoma; Li, liver; Lu, lung; Os, osteosarcoma; Pan, pancreas; Sa, sarcoma; Sto, stomach. The age at diagnosis, when known, is marked after the malignancy. The number of siblings is shown below the diamonds. For the new family #6001, (+) = mutation carrier, p53+ = positive staining in immunohistochemical analysis. The case numbers of the individuals analysed are shown below the carrier status predisposing *TP53* mutations mainly occur at specific mutation-prone regions (exons 6–7) of the conserved parts of the gene.

All 3 Finnish *TP53* germline mutations affect mutation-prone sites and have been observed previously: Tyr220Cys and Arg248Gln in LFS/LFL families (Birch et al, 1994; Varley et al, 1997b), and Asn235Ser in LFS-associated malignancies, but with an uncertain familial background of cancer predisposition (Wagner et al, 1994; Diller et al, 1995; Cornelis et al, 1997). Available data may therefore suggest that Asn235Ser could be associated with variable cancer susceptibility and reduced penetrance. Results from both our (Figure 1) and other studies indicate that these mutations may be associated in particular with female breast cancer, frequently bilateral, and young age of onset, in combination with other signatures of LFS or LFL.

In conclusion, this systematic, large-scale study of germline *TP53* alterations among breast cancer families indicates that mutations in the conserved regions of the gene seem to explain only a very small fraction of the Finnish *BRCA1* and *BRCA2* mutation-negative breast cancer cases, and that additional and more important hereditary breast cancer susceptibility genes remain to be identified.

### ACKNOWLEDGEMENTS

We gratefully acknowledge Drs Tuija Löppönen, Jaakko Leisti, Guillermo Blanco, Carl Blomqvist, other contributing clinicians, and nurses Leena Kukkola and Minna Merikivi for help in patient contacts. We also wish to thank Dr Päivi Heikkilä for immunohistochemistry data, and Marika Kujala and Kati Rouhento for skilful technical assistance. This study was supported by the University of Oulu, Oulu University Hospital, Finnish Cancer Society, Cancer Foundation of Northern Finland, Finnish Breast Cancer Group, Pirkanmaa Cancer Society, Nordic Cancer Union, Sigrid Juselius Foundation, Academy of Finland and Helsinki and Tampere University Central Hospital Research Funds.

### REFERENCES

Bell DW, Varley JM, Szydlo TE, Kang DH, Wahrer DCR, Shannon KE, Lubratovich M, Verselis SJ, Isselbacher KJ, Fraumeni JF, Birch JM, Li FP, Garber JE and Haber DA (1999) Heterozygous germ line hCHK2 mutations in Li-Fraumeni syndrome. *Science* **286**: 2528–2531

Birch JM, Hartley AL, Tricker KJ, Prosser J, Condie A, Kelsey AM, Harris M, Morris Jones PH, Binchy A, Crowther D, Craft AW, Eden OB, Evans GR, Thompson E, Mann JR, Martin J, Mitchell ELD and Santibanez-Koref MF (1994) Prevalence and diversity of constitutional mutations in the p53 gene among 21 Li-Fraumeni families. *Cancer Res* **54**: 1298–1304

Boerresen A-L, Andersen TI, Garber J, Barbier-Piraux N, Thorlacius S, Eyfjörd J, Ottestad L, Smith-Soerensen B, Hovig E, Malkin D and Friend SH (1992) Screening for germ line TP53 mutations in breast cancer patients. *Cancer Res* **52**: 3234–3236

Cho Y, Gorina S, Jeffrey PD and Pavletich NP (1994) Crystal structure of a p53 tumour suppressor-DNA complex: understanding tumorgenic mutations. *Science* **267**: 1353–1356

Claus EB, Schildkraut JM, Thompson WD and Risch NJ (1996) The genetic attributable risk of breast and ovarian cancer. *Cancer* **77**: 2318–2324

Cornelis RS, van Vliet M, van de Vijver MJ, Vasen HFA, Voute PA, Top B, Meera Khan P, Devilee P and Cornelisse CJ (1997) Three germline mutations in the TP53 gene. *Hum Mutat* **9**: 157–163

 *© 2001 Cancer Research Campaign*

Diller L, Sexsmith E, Gottlieb A, Li FP and Malkin D (1995) Germline p53 mutations are frequently detected in young children with rhabdomyosarcoma. *J Clin Invest* **95**: 1606–1611

Easton DF (1999) How many more breast cancer predisposition genes are there? *Br Cancer Res* **1**: 14–17

Eng C, Schneider K, Fraumeni JF and Li FP (1997) Third international workshop on collaborative interdisciplinary studies of p53 and other predisposing genes in Li-Fraumeni syndrome. *Cancer Epidemiol Biomark Prev* **6**: 379–383

Ganguly T, Dhulipala R, Godmilow L and Ganguly A (1998) High throughput fluorescence-based conformation-sensitive gel electrophoresis (F-CSGE) identifies six unique BRCA2 mutations and an overall low incidence of BRCA2 mutations in high-risk BRCA1-negative breast cancer families. *Hum Genet* **102**: 549–556

Hollstein M, Sidransky D, Vogelstein B and Harris CC (1991) p53 mutations in human cancer. *Science* **253**: 49–53

Huusko P, Pääkkönen K, Launonen V, Pöyhönen M, Blanco G, Kauppila A, Puistola U, Kiviniemi H, Kujala M, Leisti J and Winqvist R (1998) Evidence of founder mutations in Finnish BRCA1 and BRCA2 families. *Am J Hum Genet* **62**: 1544–1548

Huusko P, Castrén K, Launonen V, Soini Y, Pääkkönen K, Leisti J, Vähäkangas K and Winqvist R (1999) Germ-line TP53 mutations in Finnish cancer families exhibiting features of the Li-Fraumeni syndrome and negative for BRCA1 and BRCA2. *Cancer Genet Cytogenet* **112**: 9–14

Kainu T, Juo S-HH, Desper R, Schäffer AA, Gillanders E, Rozenblum E, Freas-Lutz D, Weaver D, Stephan D, Bailey-Wilson J, Kallioniemi O-P, Tirkkonen M, Syrjäkoski K, Kuukasjärvi T, Koivisto P, Karhu R, Holli K, Arason A, Johannesdottir G, Bergthorsson JT, Johannsdottir H, Egilsson V, Björk Barkardottir R, Johannsson O, Haraldsson K, Sandberg T, Holmberg E, Grönberg H, Olsson H, Borg Å, Vehmanen P, Eerola H, Heikkilä P, Pyrhönen S and Nevanlinna H (2000) Somatic deletions in hereditary breast cancers implicate 13q21 as a putative novel breast cancer susceptibility locus. *Proc Natl Acad Sci USA* **97**: 9603–9608

Lasky T and Silbergeld E (1996) p53 mutations associated with breast, colorectal, liver, lung and ovarian cancers. *Environ Health Perspectives* **104**: 1324–1331

Levine AJ (1997) p53, the cellular gatekeeper for growth and division. *Cell* **88**: 323–331

Lidereau R and Soussi T (1992) Absence of p53 germ-line mutations in bilateral breast cancer patients. *Hum Genet* **89**: 250–252

Miki Y, Swensen J, Shattuck-Eidens D, Futreal PD, Harshman K, Tavtigian S, Liu Q, Cochran C, Bennett LM, Ding W, Bell R, Rosenthal J, Hussey C, Tran T, McClure M, Frye C, Hattier T, Phelps R, Haugen-Strano A, Katcher H, Yakumo K, Gholami Z, Shaffer D, Stone S, Bayer S, Wray C, Bogden R, Dayananth P, Ward J, Tonin P, Narod S, Bristow BK, Norris FH, Helvering L, Morrison P, Rosteck P, Lai M, Barrett JC, Lewis C, Neuhausen S, Cannon-Albright L, Goldgar D, Wiseman R, Kamb A and Skolnick MH (1994) A strong candidate for the breast and ovarian cancer susceptibility gene BRCA1. *Science* **266**: 66–71

Nyström-Lahti M, Kristo P, Nicolaides NC, Chang SY, Aaltonen LA, Moisio AL, Järvinen HJ, Mecklin JP, Kinzler KW, Vogelstein B, de la Chapelle A and Peltomäki P (1995) Founding mutations and Alu-mediated recombination in hereditary colon cancer. *Nat Med* **1**: 1203–1206

Patel UA, Perry M and Crane-Robinson C (1995) Screening for germline mutations of the p53 gene in familial breast cancer patients. *Eur J Clin Invest* **25**: 132–137

Prosser J, Elder PA, Condie A, MacFadyen I, Steel CM and Evans HJ (1991) Mutations in p53 do not account for heritable breast cancer: a studey in five affected families. *Br J Cancer* **63**: 181–184

Sarantaus L*, Huusko P*, Eerola H, Launonen V, Vehmanen P, Rapakko K, Gillanders E, Syrjäkoski K, Kainu T, Vahteristo P, Krahe R, Pääkkönen K, Hartikainen J, Blomqvist C, Löppönen T, Holli K, Ryynänen M, Bützow R, Borg Å, Wasteson Arver B, Holmberg E, Mannermaa A, Kere J, Kallioniemi O-P, Winqvist R** and Nevanlinna H** (*/** = shared first/senior authorship) (2000) Multiple founder effects and geographical clustering of BRCA1 and BRCA2 families in Finland. *Eur J Hum Genet* (in press)

Shibata A, Tsai YC, Press MF, Henderson BE, Jones PA and Ross RK (1996) Clonal analysis of bilateral breast cancer. *Clin Cancer Res* **2**: 743–748

Sidransky D, Tokino T, Helzlsouer K, Zehnbauer B, Rausch G, Shelton B, Prestigiacomo L, Vogelstain B and Davidson N (1992) Inherited p53 gene mutations in breast cancer. *Cancer Res* 2984–2986

Syvänen A-C (1999) From gels to chips: “Minisequencing” primer extension for analysis of point mutations and single nucleotide polymorphisms. *Hum Mutat* **13**: 1–10

Toguchida J, Yamaguchi T, Dayton SH, Beauchamp RL, Herrera GE, Ishizaki K, Yamamuro T, Meyers PA, Little JB, Sasaki MS, Weichselbaum RR and Yandeli DW (1992) Prevalence and spectrum of germline mutations of the p53 gene among patients with sarcoma. *N Engl J Med* **320**: 1301–1308

Varley JM, Evans CGR and Birch JM (1997a) Li-Fraumeni syndrome – a molecular and clinical review. *Br J Cancer* **76**: 1–14

Varley JM, McGown G, Thorncroft M, Santibanez-Koref M, Kelsey AM, Tricker KJ, Evans GR and Birch JM (1997b) Germ-Line Mutations of TP53 in Li-Fraumeni Families: An Extended Study of 39 Families. *Cancer Res* **57**: 3245–3252

Vehmanen P, Friedman LS, Eerola H, McClure M, Ward B, Sarantaus L, Kainu T, Syrjäkoski K, Pyrhönen S, Kallioniemi O-P, Muhonen T, Luce M, Frank TS and Nevanlinna H (1997a) Low porpotion of BRCA1 and BRCA2 mutations in Finnish breast cancer families: evidence for additional susceptibility genes. *Hum Mol Genet* **6**: 2309–2315

Vehmanen P, Friedman LS, Eerola H, Sarantaus L, Pyrhönen S, Ponder ABJ, Muhonen T and Nevanlinna H (1997b) A low porpotion of BRCA2 mutations in Finnish breast cancer families. *Am J Hum Genet* **60**: 1050–1058

Wagner J, Portwine C, Rabin K, Leclerc JM, Narod SA and Malkin D (1994) High frequency of germline p53 mutations in childhood adrenocortical cancer. *J Natl Cancer Inst* **86**: 1707–1710

Warren W, Eeles RA, Ponder BAJ, Easton DF, Averill D, Ponder MA, Anderson K, Evans AM, DeMars R, Love R, Dundas S, Stratton MR, Trowbridge P, Cooper CS and Peto J (1992) No evidence for germline mutations in exons 5–9 of the p53 gene in 25 breast cancer families. *Oncogene* **7**: 1043–1046

Wooster R, Bignell G, Lancaster J, Swift S, Seal S, Mangion J, Collins N,Gregory S, Gumbs C, Micklem G, Barfoot R, Hamoudi R, Patel S, Rice C, Biggs P, Hashim Y, Smith A, Connor F, Arason A, Gudmundsson J, Ficenec D, Kelsell D, Ford D, Tonin P, Bishop DT, Spurr NK, Ponder BAJ, Eeles R, Peto J, Devilee P, Cornelisse C, Lynch H, Narod S, Lenoir G, Egilsson V, Barkardottir RB, Easton DF, Bentley DR, Futreal PA, Ashworth A and Stratton MR (1995) Indentification of the breast cancer susceptibility gene BRCA2. *Nature* **378**: 789–792

Zelada-Hedman M, Boerresen-Dale A-L, Claro A, Chen J, Skoog L and Lindblom A (1997) Screening for TP53 mutations in patients and tumours from 109 Swedish breast cancer families. *Br J Cancer* **75**: 1201–1204

*© 2001 Cancer Research Campaign*

